# Recent studies on ursolic acid and its biological and pharmacological activity

**DOI:** 10.17179/excli2016-159

**Published:** 2016-03-14

**Authors:** Sook Young Lee, Yong Joo Kim, Sun Ok Chung, Sang Un Park

**Affiliations:** 1 Regional Innovation Center for Dental Science and Engineering, Chosun University, 309 Pilmun-daero, Dong-gu, Gwangju, 501-759, Korea; 2 Department of Biosystems Machinery Engineering, Chungnam National University, 99 Daehak-ro, Yuseong-gu, Daejeon, 305-764, Korea; 3 Department of Crop Science, Chungnam National University, 99 Daehak-ro, Yuseong-gu, Daejeon, 305-764, Korea

## Dear Editor,

Ursolic acid (3-beta-3-hydroxy-urs-12-ene-28-oic-acid; UA) is a lipophilic pentacyclic triterpenoid; it was found to be present in the epicuticular waxes of apples in 1920. It is widely found naturally in the peels of fruits, as well as in many herbs and spices such as lavender, oregano, thyme, rosemary, and thyme (Woźniak et al., 2015[48]). UA has been confirmed to have several biological and pharmacological effects, such as anti-inflammatory (Baricevic et al., 2001[6]), antitumor (Baglin et al., 2003[3]), antiplatelet aggregation (Babalola et al., 2013[1]), anti-HIV (Kashiwada et al., 2000[25]), and anti-*Mycobacterium tuberculosis* effects (Cantrell et al., 2001[7]).

Its many pharmaceutical and biological properties make it an interesting material for application in the pharmaceutical, food, and cosmetics industries. Herein, we review the most recent studies on UA and its biological and pharmacological activities (Table 1(Tab. 1)). (References in Table 1: Meng et al., 2015[37]; He et al., 2015[17]; Wang et al., 2015[46]; Yang et al., 2015[51]; Chu et al., 2015[11]; Jeon et al., 2015[21]; Cho et al., 2015[10]; Yuliang et al., 2015[54]; Bakhtiari et al., 2015[4]; Prissadova et al., 2015[41]; Yuan et al., 2015[53]; Li et al., 2015[30]; Xu et al., 2015[50]; Xiang et al., 2015[49]; Jiang et al., 2015[23]; Gao et al., 2015[15]; Kim and Moon, 2015[26]; Yie et al., 2015[52]; Kim et al., 2015[28]; Chen et al., 2015[9]; Ma et al., 2015[36]; Hu et al., 2015[19]; Jia et al., 2015[22]; Podder et al., 2015[40]; Zhang et al., 2014[55]; Zou et al., 2014[57]; Weng et al., 2014[47]; Kalani et al., 2014[24]; Hwang et al., 2014[20]; Bang et al., 2014[5]; He et al., 2014[18]; Castro et al., 2015[8]; Liu et al., 2013[34]; Sundaresan et al., 2014[44]; Baek et al., 2014[2]; Lee et al., 2014[29]; Saini et al., 2014[42]; Chun et al., 2014[12]; Ou et al., 2014[39]; Li et al., 2014[31]; Ma et al., 2014[35]; Qu et al., 2013; Han et al., 2014[16]; Kim et al., 2014[27]; Deng et al., 2014[13]; Ling et al., 2013[33]; do Nascimento et al., 2014[14]; Wang et al., 2013[45]; Nam and Kim et al., 2013[38]; Lin et al., 2013[32]; Song et al., 2014[43]; Zhou et al., 2013[56])

## Acknowledgements

This research was supported by Agriculture, Food and Rural Affairs Research Center Support Program, Ministry of Agriculture, Food and Rural Affairs.

## Conflict of interest

The authors declare no conflict of interest.

## Figures and Tables

**Table 1:**
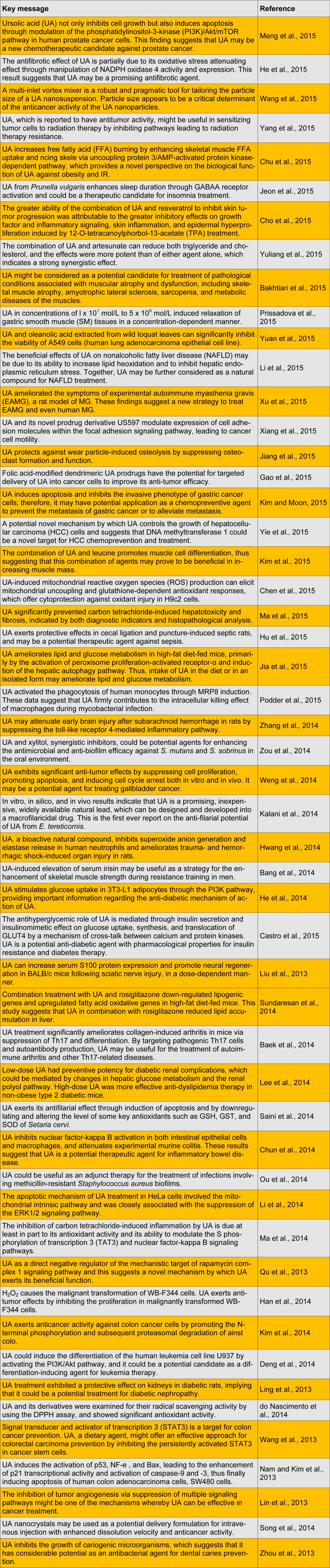
Recent studies on ursolic acid and its biological and pharmacological activities
